# The Efficacy of Local Insulin Versus Topical Phenytoin or Normal Saline in Diabetic Foot Ulcer Management: A Prospective Comparative Study

**DOI:** 10.7759/cureus.30461

**Published:** 2022-10-19

**Authors:** Jamuna Nagaraj, Venkatesh Subbiah

**Affiliations:** 1 General Surgery, Dhanalakshmi Srinivasan Medical College and Hospital, Siruvachur, IND; 2 General Surgery, Velammal Medical College and Hospital, Madurai, IND

**Keywords:** wound healing, local injectable insulin, normal saline dressing, topical phenytoin, diabetes foot ulcer

## Abstract

Introduction

During an individual's lifetime, around 15% to 25% of patients with diabetes mellitus develop foot ulcers, and about 1% of patients end up with an amputation. For the past two decades, we have treated diabetic foot ulcers with a variety of methods including cleansing and dressing after debridement of the lesion, where the dressing is applied using local insulin, topical phenytoin, normal saline dressing, etc. We conducted the present study to compare the efficacy of diabetic foot ulcer management between local injectable insulin, topical phenytoin, and normal saline among diabetic patients.

Methodology

We conducted a prospective study of 60 patients with diabetic foot ulcers who sought outpatient care at the Department of General Surgery, in a tertiary care hospital in Perambalur, from September 2021 to August 2022. We included all patients who provided informed written consent and had ulcers ranging from grade 1-2. We excluded patients with foot ulcers caused by other etiologies such as osteomyelitis and renal failure. We divided the selected study participants into three groups using the number lot method (randomization). Twenty study participants made up each group; there were three groups in total - groups 1, 2, and 3. Group 1 was treated with local insulin; group 2 with topical phenytoin; and group 3 with normal saline dressing. Before the start of the study, we measured the wound size and depth of the wound and followed up at seven days intervals for one month. The results were analyzed using SPSS, version 21 (IBM Corp., Armonk, NY).

Results

The mean age, duration of diabetes, and mean fasting blood sugar values of the study participants in the three groups were almost similar. The mean difference in wound size before and after treatment in the insulin, normal saline, and phenytoin groups was 4.98, 3.74, and 3.805 square centimeters, respectively. This difference in mean among the above three groups was statistically significant (P < 0.001). The mean difference in wound depth before and after treatment in the insulin, normal saline, and phenytoin groups was 47.005, 4.945, and 4.820 square centimeters, respectively. This difference in mean among the above three groups was statistically significant (P < 0.001). Thus, wound healing was better in the local insulin group than in the other two groups, with statistical significance. The mean number of days taken for wound healing in the insulin, normal saline, and phenytoin groups was 20, 26, and 23 days, respectively. This difference in mean among the above three groups was statistically significant (P < 0.001).

Conclusion

The current study, which lasted a year in a tertiary care hospital, found that local injectable insulin heals diabetic foot ulcers more rapidly than local topical phenytoin, which is superior to the standard treatment of using normal saline.

## Introduction

Diabetes is a chronic, metabolic disease characterized by elevated levels of blood glucose (or blood sugar), which leads to serious damage over time as it causes microvascular and macrovascular complications. The most common is type 2 diabetes, usually in adults, which occurs when the body becomes resistant to insulin or does not make enough insulin. In the past three decades, the prevalence of type 2 diabetes has risen dramatically in countries of all income levels. There is a globally agreed target to halt the rise in diabetes and obesity by 2025 [1]. One-fifth of the world's population comprises South Asians, who have a considerably increased incidence of type-2 diabetes mellitus (T2DM). Compared to the overall population, South Asians have a 50% greater prevalence of diabetes [2].

Diabetic foot ulcers are among the most common complications of uncontrolled diabetes. About 5% of patients with diabetes mellitus develop foot ulcers, and 1% end up with amputation [3]. In India, diabetic foot ulcers are the common cause of non-traumatic amputation of limbs, which is preventable [4]. It is mainly because of ineffective glycemic control and inappropriate foot care, along with the underlying neuropathy [5]. Worldwide, the yearly incidence of diabetic foot ulcers ranges between 9.1 and 26.1 million [6].

Around 15% to 25% of patients with diabetes mellitus develop a diabetic foot ulcer during their lifetime. As the number of newly diagnosed diabetics is increasing yearly, the incidence of diabetic foot ulcers is also bound to increase [7]. Management of diabetic foot ulcers should be systematic to achieve the best results. At the initial stage of the presentation, it is better to identify and rule out other potential causes of the presenting complaint. Remarkably, severe diabetic foot infection can also cause minimal systemic signs of infection [8].

Management of the diabetic foot ulcer itself is a complicated process as it takes a long time for evaluation and treatment and for the response to the treatment to occur. It takes a longer time for the formation of granulation tissue. Diabetic foot ulcers have been treated with a variety of methods including cleansing and dressing after debridement of the lesion, where the dressing is applied using local insulin, topical phenytoin, normal saline dressing, etc. Normal saline is used since it is sterile, cost-effective, and provides moisture which augments wound healing. As per earlier studies, the anti-serine-threonine kinase and anti-phosphor extracellular signal-regulated protein kinase pathways are enhanced by local insulin injection which speeds up the healing process. Phenytoin, an anti-epileptic drug, enhances the healing process by improving collagen deposition and also helps in the formation of granulation tissue [9,10].

With this background, we conducted the present study among diabetic patients to compare the efficacy of diabetic foot ulcer management between local injectable insulin, topical phenytoin, and normal saline.

## Materials and methods

Study design and setting

We conducted a randomized prospective study to compare the efficacy of local insulin versus topical phenytoin in the treatment of diabetic foot ulcers among the diabetic patients attending the clinic at the Department of General Surgery in a tertiary care hospital, Perambalur. We conducted this study after getting ethical clearance from the institutional ethics committee (Approval No: IECHS/IRCHS/No.174).

Study population and duration

The study comprised 60 patients with diabetic foot ulcers who sought outpatient care at a tertiary care hospital for the course of one year, from September 2020 to August 2021.

Inclusion and exclusion criteria

The Wagner-Meggitt classification of diabetic feet was used to diagnose diabetic foot ulcers up to grade 2 in all patients who provided informed written consent. According to the Wagner-Meggitt classification, ulcers were classified as grade 0 - intact skin; grade 1 - superficial ulcer of the skin or subcutaneous tissue; grade 2 - deep ulcers that extend into the bone, ligament, or joint.

The study excluded patients with foot ulcers caused by other etiologies such as osteomyelitis and renal insufficiency.

Randomization

Using the number lot approach, we separated the chosen study participants into three groups. Twenty study participants made up each group which was designated Groups 1, 2, and 3.

Group 1 had their diabetic ulcers treated with local insulin (10 IU for each square centimetre of the wound); Group 2 had their diabetic ulcers treated with topical phenytoin; Group 3 had their diabetic foot ulcers treated with normal saline dressing, i.e., conventional treatment.

Study procedure

At the start of the study, the complete demographic details of the patients were recorded, and the recorded details included age, sex, duration of diabetes mellitus, treatment, peripheral pulses, and neuropathic changes. All the routine investigations of the patient were done. Before the study, we brought in strict glycemic control for all patients. We provided the antibiotic after doing a culture and sensitivity test on the entire site and did surgical debridement of the wound as per the wound.

We evaluated the wound’s size and depth before the investigation began, and continued to take measures regularly with a seven-day interval between them. Follow-up measures were taken for four weeks throughout the trial. We calculated the required dose of insulin for the study subjects in Group 1 - it is diluted to 1 ml with distilled water, and injected directly into the base of the wound. In Group 2, topical phenytoin was used for cleaning and dressing the wound. In Group 3, normal saline was used for dressing the wound.

Data entry and analysis

The data obtained were entered into Microsoft Excel. We analyzed the results using SPSS version, 21 (IBM Corp., Armonk, NY). To determine the relationship between wound size and depth and the three care techniques, an analysis of variance (ANOVA) test was performed. By using the chi-square test, the relationship between the three management techniques and the development of granulation tissue was determined. A P-value of less than 0.05 was considered statistically significant.

## Results

We created three groups totaling 60 individuals: an insulin group, a normal saline group, and a phenytoin group. Table 1 describes the general characteristics of the study participants among the insulin, normal saline, and phenytoin groups. The mean age, duration of diabetes, and mean fasting blood sugar values of the study participants in the three groups were almost similar. The mean average number of days taken for wound healing was lower (20 days) in the insulin group than in the phenytoin group (23 days), which is better than the normal saline group (26 days). The mean size of the wound after treatment in the phenytoin group and the normal saline group was 3.56 and 3.96 square centimeters, respectively; the mean size of the wound in the insulin group reduced better (2.3 square centimeters). The mean depth of the wound after the treatment was 2.455 millimeters, which was better than the phenytoin group and normal saline group (4.13 and 4.425 millimeters, respectively).

**Table 1:** Description of characteristics of the study participants between the three groups - insulin group (n = 20), normal saline group (n = 20), and phenytoin group (n = 20)

S. No	Variables		Insulin group	Normal saline group	Phenytoin group
1	Age (in years)	Mean	55.60	54.95	55.55
		Standard Deviation	7.783	10.575	10.768
2	Duration of diabetes (in years)	Mean	11.40	12.40	11.30
		Standard Deviation	3.283	4.044	3.389
3	Mean Fasting Blood Sugar (in mg/dl)	Mean	101.65	99.65	101.40
		Standard Deviation	18.576	23.272	20.712
4	Average number of days took of wound healing	Mean	20.35	26.05	22.90
		Standard Deviation	2.084	3.103	3.161
5	Size of wound in before treatment (in cm^2^)	Mean	7.330	7.705	7.370
		Standard Deviation	0.504	1.028	0.893
6	Size of wound in after treatment (in cm^2^)	Mean	2.345	3.960	3.565
		Standard Deviation	0.397	0.625	0.507
7	Depth of the wound before treatment (in mm)	Mean	9.460	9.370	8.950
		Standard Deviation	1.614	1.520	1.890
8	Depth of the wound after treatment (in mm)	Mean	2.455	4.425	4.130
		Standard Deviation	0.432	0.525	0.733

Table 2 describes the gender distribution of the study participants among the insulin, normal saline, and phenytoin groups. Males made up 65%, 70%, and 55% of the insulin, normal saline, and phenytoin groups, respectively. The proportion of males was higher in the normal saline group than in the insulin and phenytoin groups.

**Table 2:** Gender distribution of the study participants in insulin, normal saline, and phenytoin groups - insulin group (n = 20), normal saline group (n = 20), and phenytoin group (n = 20)

Group	Gender	Frequency	Percent
Insulin group	Female	7	35.0
	Male	13	65.0
	Total	20	100.0
Normal saline group	Female	6	30.0
	Male	14	70.0
	Total	20	100.0
Phenytoin group	Female	9	45.0
	Male	11	55.0
	Total	20	100.0

Table 3 describes the formation of granulation tissue during follow-up among the study participants in the insulin, normal saline, and phenytoin groups. At the end of the first week, we classified all the participants in three groups as poor in terms of the formation of granulation tissue. The proportion of participants classified as satisfactory was in an increasing trend during the follow-up at the end of the 2nd, 3rd, and 4th weeks, which was 10%, 45%, and 75%, respectively. This proportion was better than (quicker formation of granulation tissue) the normal saline group and the phenytoin group.

**Table 3:** Distribution of study participants among insulin, normal saline, and phenytoin groups according to formation of granulation tissue - insulin group (n = 20), normal saline group (n = 20), and phenytoin group (n = 20)

S. No	Follow-up after treatment	Group	Formation of granulation tissue	Frequency	Percent
1	At the end of 1st week	Insulin group	Poor	20	100.0
		Normal saline group	Poor	20	100.0
		Phenytoin group	Poor	20	100.0
2	At the end of 2nd week	Insulin group	Poor	14	70.0
			Healthy	2	10.0
			Satisfactory	4	20.0
		Normal saline group	Poor	19	95.0
			Satisfactory	1	5.0
		Phenytoin group	Poor	18	90.0
			Satisfactory	2	10.0
3	At the end of 3rd week	Insulin group	Poor	2	10.0
			Healthy	9	45.0
			Satisfactory	9	45.0
		Normal saline group	Poor	10	50.0
			Satisfactory	10	50.0
		Phenytoin group	Poor	7	35.0
			Satisfactory	13	65.0
4	At the end of 4th week	Insulin group	Healthy	15	75.0
			Satisfactory	5	25.0
		Normal saline group	Poor	8	40.0
			Healthy	3	15.0
			Satisfactory	9	45.0
		Phenytoin group	Poor	4	20.0
			Healthy	4	20.0
			Satisfactory	12	60.0

Table 4 describes the association of wound size, depth, and number of days of wound healing between insulin, phenytoin, and the normal saline group. The mean difference in wound size before and after treatment in the insulin, normal saline, and phenytoin groups was 4.98, 3.74, and 3.805 square centimeters, respectively. This difference in mean among the above three groups was statistically significant according to the ANOVA test (P < 0.001). The mean difference in wound depth before and after treatment in the insulin, normal saline, and phenytoin groups was 7.005, 4.945, and 4.820 millimeters, respectively. This difference in mean among the above three groups was statistically significant according to the ANOVA test (P < 0.001). Thus, wound healing was better in the local insulin group than in the other two groups, with statistical significance. The mean number of days taken for wound healing in the insulin, normal saline, and phenytoin groups was 20, 26, and 23 days, respectively. This difference in mean among the above three groups was statistically significant according to the ANOVA test (P < 0.001). Thus, the wound healing was quicker in the local insulin group than in the other two groups.

**Table 4:** Association of wound size, depth, and number of days of wound healing between insulin, phenytoin, and normal saline group - insulin group (n = 20), normal saline group (n = 20), and phenytoin group (n = 20) ^*^Obtained by analysis of variance (ANOVA) test

S. No	Variables		Insulin group	Normal saline group	Phenytoin group	Table Value^*^	P-Value
1	Difference in wound size before and after treatment	Mean	4.985	3.745	3.805	11.919	< 0.001
		Std. Deviation	0.604	1.190	0.826		
2	Difference in the depth of the wound before and after treatment	Mean	7.005	4.945	4.820	9.749	< 0.001
		Std. Deviation	1.711	1.653	1.134		
3	Number of days took of wound healing	Mean	20.35	26.05	22.90	20.413	< 0.001
		Std. Deviation	2.084	3.103	3.161		

Table 5 describes the association of granulation tissue formation between insulin, phenytoin, and the normal saline group. There is no statistical significance in the 2nd week of the treatment. But, at the 3rd and 4th weeks of treatment, there was a statistically significant association between the local insulin group and the formation of granulation tissue (P < 0.001) when compared to phenytoin and the normal saline group; we got this statistical significance using the chi-square test.

**Table 5:** Association of granulation tissue formation between insulin, phenytoin, and normal saline groups - insulin group (n = 20), normal saline group (n = 20), and phenytoin group (n = 20) *Fischer exact test

S.no	Follow-up	Group		Formation of granulation tissue			P-Value
				Healthy	Poor	Satisfactory	
1	At the end of 2^nd^ week of treatment	Insulin group	n	2	14	4	0.205
			%	10.0%	70.0%	20.0%	
		Normal saline group	n	0	19	1	
			%	0.0%	95.0%	5.0%	
		Phenytoin group	n	0	18	2	
			%	0.0%	90.0%	10.0%	
2	At the end of a 3^rd^ week of treatment	Insulin group	n	9	2	9	< 0.001
			%	45.0%	10.0%	45.0%	
		Normal saline group	n	0	10	10	
			%	0.0%	50.0%	50.0%	
		Phenytoin group	n	0	7	13	
			%	0.0%	35.0%	65.0%	
3	At the end of a 4^th^ week of treatment	Insulin group	n	15	0	5	< 0.001
			%	75.0%	0.0%	25.0%	
		Normal saline group	n	3	8	9	
			%	15.0%	40.0%	45.0%	
		Phenytoin group	n	4	4	12	
			%	20.0%	20.0%	60.0%	

## Discussion

According to the current study, local injectable insulin has higher efficacy than local phenytoin, which is also comparatively more effective than normal saline in treating diabetic foot ulcers.

In our study, the average number of days required for wound healing was 20 days in the insulin group, 26 days in the normal saline group, and 22 days in the phenytoin group. And it was found that there was a statistically significant association between rapid wound healing with local insulin and the other two methods. It was found that there was a statistically significant association between wound healing in terms of wound size with local insulin compared to other treatment methods. Similarly, It was found that there was a statistically significant association in wound healing in terms of wound depth with local insulin compared to other treatment methods. Also, our study found that there was a statistically significant association in wound healing in terms of the formation of granulation tissue with local insulin than with other treatment methods in the 3rd and 4th week after the treatment.

Zhang et al. conducted a prospective study in 2016 in China with 32 patients who had diabetic foot ulcers to determine the impact of insulin on the development of granulation tissue. In contrast to our study results, the above study found that the granulation tissue formation occurred at seven days and it was statistically significant when compared to normal saline treatment; [11] this requires further exploration.

Our study results were also supported by Martínez-Jiménez et al. who conducted research in 2013 with eight diabetic patients who had foot ulcers to evaluate the impact of local insulin, and the findings were comparable. This study concluded that there was a statistically significant improvement in the formation of new blood vessels around the ulcer site and granulation tissue which enables wound healing [12].

Similar to our study results, one more study by Bhettani et al., in 2019 found a statistically significant association between wound healing and local insulin treatment among 110 patients diagnosed with diabetic foot ulcers. This study aimed to compare the efficacy of local insulin versus placebo in treating diabetic ulcers. They found that the wound diameter was reduced in the insulin group when compared to the placebo group with a statistical significance of p = 0.022 [13].

The investigation carried out by Rao et al. produced findings that were comparable to those found in the current study [5]. They reported that wound healing is better with local injectable insulin with a statistical significance. And they also found that the reduction in size and depth of the wound was around 79.4% when treated with local insulin, 59.3% when treated with local phenytoin, and 40% with conventional treatment. They reduced the depth of the wound to 77.7% with local insulin, 69% with local phenytoin, and 51.2% with conventional treatment.

A study conducted by Greenway et al. reported that the percentage change in the mean wound surface area of the wound in the group treated with phenytoin was 59.3%, which is less than the group treated with local insulin. This outcome was comparable to our study’s finding that phenytoin is more effective than regular saline in treating diabetic foot ulcers [14]. We compared this finding with a study result conducted by Sanjay et al. In that study, before treatment, the mean size of the wound among the study participants with local insulin was 4.8 cm2, and the group treated with normal saline was 4.9 cm2. After treatment with local insulin and normal saline, the size of the foot ulcer was reduced to 2.1 cm2 and 4.5 cm2 respectively, which showed the effectiveness of local insulin [15].

Limitations

This study was done with a small sample size in each group, which might influence the generalizability. The adverse effects of the therapy process such as systemic impacts were not gathered or examined in this study. Also, the concurrent systemic medication taken by the patients that may impact wound healing was not considered. Our study did not conduct a regression analysis, thus we could not estimate the impact of the risk factors. A multi-centric study with more sample size may yield better results.

## Conclusions

The current study, which lasted a year in a tertiary care hospital, found that local injectable insulin heals diabetic foot ulcers more rapidly than local topical phenytoin, which is superior to the standard treatment of using normal saline. Local injectable insulin was more effective in the short-term healing of diabetic foot ulcers than topical phenytoin and regular saline. Thus, surgeons can opt for local insulin to treat diabetic foot ulcers.
